# Measurement of overweight and obesity an urban slum setting in sub-Saharan Africa: a comparison of four anthropometric indices

**DOI:** 10.1186/s40608-016-0126-0

**Published:** 2016-11-08

**Authors:** Tilahun Nigatu Haregu, Samuel Oti, Thaddaeus Egondi, Catherine Kyobutungi

**Affiliations:** 1 African Population and Health Research Center, P.O. Box 10787 code 00100, Nairobi, Kenya; 2 International Development Research Centre, PO Box 62084-00200, Nairobi, Kenya; 3 Drugs for Neglected Diseases initiative (DNDi) Africa, P.O. Box 21936-00505, Nairobi, Kenya

**Keywords:** Anthropometry, Overweight, Obesity, Africa

## Abstract

**Background:**

As a result of both genetic and environmental factors, the body composition and topography of African populations are presumed to be different from western populations. Accordingly, globally accepted anthropometric markers may perform differently in African populations. In the era of rapid emergence of cardio-vascular diseases in sub-Saharan Africa, evidence about the performance of these markers in African settings is essential. The aim of this study was to investigate the inter-relationships among the four main anthropometric indices in measuring overweight and obesity in an urban poor African setting.

**Methods:**

Data from a cardiovascular disease risk factor assessment study in urban slums of Nairobi were analyzed. In the major study, data were collected from 5190 study participants. We considered four anthropometric markers of overweight and obesity: Body Mass Index, Waist Circumference, Waist to Hip Ratio, and Waist to Height Ratio. Pairwise correlations and kappa statistics were used to assess the relationship and agreement among these markers, respectively. Discordances between the indices were also analyzed.

**Results:**

The weighted prevalence of above normal body composition was 21.6 % by body mass index, 28.9 % by waist circumference, 45.5 % by waist to hip ratio, and 38.9 % by waist to height ratio. The overall inter-index correlation was +0.44. Waist to hip ratio generally had lower correlation with the other anthropometric indices. High level of discordance exists between body mass index and waist to hip ratio. Combining the four indices shows that 791 (16.1 %) respondents had above normal body composition in all four indices. Waist circumference better predicted hypertension and hyperglycemia while waist to height ratio better predicted hypercholesterolemia.

**Conclusions:**

There exists a moderate level of correlation and a remarkable level of discordance among the four anthropometric indices with regard to the ascertainment of abnormal body composition in an urban slum setting in Africa. Waist circumference is a better predictor of cardio-metabolic risk.

## Background

*Globesity* –a term that describes the escalating global epidemic of overweight and obesity– appears to be catching up with Sub-Saharan Africa (SSA) [1, 2]. According to the 2013 Global Burden of Disease (GBD) study, obesity is already a major public health challenge in many middle income countries. For example, Kenya recently became a middle income country and it was estimated that in 2013 about 36 and 49 % of men and women aged 20 years or older respectively were either overweight or obese. The GBD study goes further to state that ‘tracking this important risk to health with increased precision and disaggregation in both developing and developed countries is a key global health priority’ [3].

Body mass index (BMI) has been utilized globally as a practical low-cost objective measure for tracking obesity, and large pooling studies have shown consistent increased risk for cardiometabolic disease and other chronic conditions as BMI reached more than 23 kg/m^2^ [4–7]. However, as an indirect measure of body fat, BMI has various drawbacks as a measure of obesity. BMI may not reflect the body changes that occur with age. The proportion of body fat increases with age, whereas muscle mass decreases, but corresponding changes in height, weight and BMI may not reflect changes in body fat and muscle mass [8].

Intra-abdominal fat has been identified as being the most clinically relevant type of fat in humans. Correspondingly, an assessment of body-fat distribution could possibly identify subjects with the highest risk of adverse lipid profile and hypertension [9]. In white adults, higher waist circumference was positively associated with higher mortality at all levels of BMI from 20 to 50 kg/m2. Therefore, it is recommended that waist circumference should be used in combination with BMI, even for those in the normal BMI range, as part of risk assessment for obesity-related premature mortality [10].

Some studies show that Waist to Height Ratio (WHtR) had significantly greater discriminatory power compared with BMI. Compared with BMI, Waist Circumference (WC) improved discrimination of adverse outcomes by 3 % and WHtR improved discrimination by 4–5 % over BMI. Studies have also shown that WHtR to be significantly better than WC for diabetes, hypertension, and other Cardiometabolic Disease (CMD) outcomes in men and women [11].

Waist circumference, BMI and Waist to Hip ratio (WHR) identified different proportions of the population, as measured by both prevalence of obesity and CVD risk factors. Whilst WHR had the strongest correlations with CVD risk factors before adjustment for age, the three obesity measures performed similarly after adjustment for age. Given the difficulty of using age-adjusted associations in the clinical setting, these results suggest that given appropriate cut-off points, WHR is the most useful measure of obesity to use to identify individuals with CVD risk factors [12].

One of the key challenges in the use of anthropometric indices has been the variation in cut-off points. A study conducted in Benin and Haiti reported optimal WC cut-offs to be 80 cm and 94 cm in men and women, respectively, which was exactly the reverse of the generic cut-offs. In that study, the standard 0.50 cut-off of WHtR appeared valid for men, but it had to be increased to 0.59 in women [13]. In the Chinese population, the optimal cut-off values were approximately 24 · 0 and 23 · 0 kg/m2 for BMI, 85 · 0 and 75 · 0 cm for WC, and 0 · 50 and 0 · 48 for WHtR for men and women, respectively [14]. In Tunisia, BMI cut-off points were 24 kg/m(2) in men and 27 kg/m(2) in women while that of WC were 85 cm in men and 85 cm in women [15]. In South African population, the appropriate waist cut point for diagnosing metabolic syndrome was found to be 91.5 cm [16].

With regard to the interrelationships among anthropometric indices, there are a remarkable number of studies conducted in African Americans [17–19]. However, as a result of both genetic and environmental factors, the body composition and architecture of African populations living in Africa is presumed to be different from western populations [20]. Accordingly, anthropometric markers may perform differently in these populations. In the era of rapid emergence of cardio-metabolic diseases in sub-Saharan Africa, evidence about the performance of these markers in African settings is essential [21].

Hence, the objective of this study was to investigate the inter-relationships among the four main anthropometric indices (Body Mass Index, Waist Circumference, Waist to Hip Ratio, and Waist to Height Ratio) in measuring overweight and obesity in an urban poor setting in Sub-Saharan African.

## Methods

### Data source

This study utilized data collected as part of a population-based cross-sectional survey designed to assess the linkages between socioeconomic and sociocultural factors, perceived personal risk for cardiometabolic disease, and health behavior in the *Korogocho* and *Viwandani* slums of Nairobi. The survey was conducted between May 2008 and April 2009 within the Nairobi Urban and Health Demographic Surveillance System (NUHDSS), which is run by the African Population and Health Research Center (APHRC). Detailed methodology of the NUHDSS is reported elsewhere [22, 23]. A total of 5190 residents of the two slums were randomly selected and stratified by sex and age using a sampling frame that included all adults aged 18 years or older in the NUHDSS. Overall response rates were 94 % in *Korogocho* and 95 % in *Viwandani*. More details of the sampling and data collected are reported elsewhere [20].

### Measurements

The weight of each respondent was measured by trained field interviewers to the nearest 0.1 kg using a SECA electronic digital weighing scale. Height was measured to the nearest cm using SECA electronic digital weighing scale and a SECA portable stadiometer (Seca GmbH, Hamburg, Germany). Waist and hip circumference were measured in centimeters using an inelastic tape measure (Figure Finder) placed directly on the skin at the level of the iliac crest (for waist) and at the maximum extension of the buttocks (for hip) [24]. The reliability of all measurements between field interviewers was assessed during training and piloting of the questionnaire. All the anthropometric measurements were done twice and an average of these two measurements was used in the analysis.

BMI was calculated as body weight in Kg divided by the square of the height in meters (17). WHR and WHtR were calculated by dividing waist circumference by hip circumference and height, respectively. Most commonly used cut-off points (BMI - 25 kg/m^2^, WC-90 cm for men and 80 cm for women, WHR −0.90 for men and 0.85 for women, and WHtR – 0.5) were used for these anthropometric indices [25–27].

Three blood pressure measurements were conducted using OMRON M6 blood pressure machine. The average of the second and the third blood pressure measurements were used in the analysis. ACCUCHECK GCT monitors and test strips were used for the measurement of random blood sugar, total blood cholesterol and triglyceride levels. Blood pressure levels greater than 140/90mmhg (or on treatment), random blood sugar >140 mg.dl, and total blood cholesterol levels >200 mg/dl were considered as increased levels of biomarkers.

### Statistical analysis

Descriptive analyses of BMI, WC, WHR, and WHtR by age and sex were conducted by using means, standard deviations and proportions. The proportions of respondents who were overweight/obese were calculated for both sexes using the four indices. Association and agreements among anthropometric indices were examined using pairwise correlations and kappa statistics, respectively. Discordances between the indices were analyzed using cross-tabulations. The four anthropometric indices were also associated with key cardiometabolic risk factors including hypertension, hyperglycemia and hypercholesterolemia. Results of these associated were presented using odds ratio (adjusted for age and sex) and its 95 % confidence intervals.

## Results

### Background characteristics

A total of 5, 190 study participants above 18 years of age were included in this analysis. Of these 2794 (53.8 %) were men while the rest 2396 (46.2 %) were women. The majority, 2899 (55.8 %) of the study population were below the age of 45 years while 673 (13 %) were above 60 years of age.

### Body mass index (BMI)

The mean (SD) BMI (in kg/m^2^) of the study population was 23.47 (4.3). Women had a significantly higher mean BMI as compared to men and an increasing trend of body mass index across age groups was observed. Women in the age group of 45–60 years had higher BMI as compared to all age sex categories as described in Table 1.

**Table 1:** Mean (SD) Anthropometric measures and indices by age-sex categories

	Age groups	Men	Women	Total
Weight (Kg)	Less than 45 years	60.82 (8.45)	62.07 (12.11)	61.44 (10.42)
	45–60 years	63.00 (10.03)	65.43 (13.62)	63.98 (11.68)
	Above 60 years	62.17 (10.57)	62.21 (12.98)	62.19 (11.68)
	All age groups	61.74 (9.36)	63.02 (12.74)	62.33 (11.05)
Height (cm)	Less than 45 years	166.93 (7.41)	159.28 (6.62)	163.16 (8.00)
	45–60 years	167.37 (7.00)	158.71 (7.17)	163.83 (8.25)
	Above 60 years	165.16 (7.57)	156.45 (7.51)	161.20 (8.70)
	All age groups	166.85 (7.32)	158.77 (6.95)	163.13 (8.21)
Hip cir. (cm)	Less than 45 years	89.49 (8.03)	97.38 (10.95)	93.34 (10.35)
	45–60 years	91.67 (9.20)	100.82 (11.74)	95.38 (11.24)
	Above 60 years	90.91 (9.79)	98.42 (13.65)	94.30 (12.26)
	All age groups	90.41 (8.74)	98.46 (11.63)	94.10 (10.92)
BMI	Less than 45 years	21.85 (2.92)	24.40 (4.57)	23.13 (4.04)
	45–60 years	22.50 (3.34)	25.98 (5.36)	23.92 (4.61)
	Above 60 years	22.76 (3.64)	25.41 (4.98)	23.93 (4.48)
	All age groups	22.19 (3.2)	24.99 (4.9)	23.47 (4.30)
Waist Circumference (cm)	Less than 45 years	78.94 (8.12)	83.40 (11.03)	81.12 (9.89)
	45–60 years	83.34 (9.43)	89.04 (11.68)	85.65 (10.77)
	Above 60 years	84.58 (9.98)	88.69 (12.72)	86.45 (11.48)
	All age groups	81.16 (9.14)	85.62 (11.74)	83.2 (10.65)
Waist to Hip Ratio	Less than 45 years	0.88 (0.06)	0.86 (0.75)	0.87 (0.07)
	45–60 years	0.91 (0.06)	0.88 (0.07)	0.89 (0.06)
	Above 60 years	0.93 (0.07)	0.90 (0.07)	0.92 (0.07)
	All age groups	0.89 (0.06)	0.87 (0.08)	0.88 (0.07)
Waist to Height Ratio	Less than 45 years	0.47 (0.05)	0.52 (0.07)	0.49 (0.06)
	45–60 years	0.49 (0.05)	0.56 (0.07)	0.52 (0.07)
	Above 60 years	0.51 (0.06)	0.57 (0.08)	0.54 (0.08)
	All age groups	0.48 (0.06)	0.54 (0.07)	0.51 (0.07)

The overall prevalence of high BMI (≥25 kg/m^2^) was found to be 28.3 %. Women had significantly higher prevalence of increased BMI than men (42.6 % versus 16.3 %). The prevalence of increased BMI increased by age. In the total study population, the prevalence of overweight (BMI ≥25 kg/m^2^ but less than 30 kg/m^2^) obesity (BMI ≥ 30 kg/m^2^) were 20.45 % and 7.86 %, respectively. The prevalence of obesity was 15.05 % in women and 1.78 % in men. Those in the age range of 45–60 years had the highest prevalence of obesity (9.94 %). In the study population, 384 (7.7 %) were found to be underweight (BMI < 18 kg/m^2^).

### Waist circumference (WC)

The mean (SD) waist circumference of the study population was 83.2 (10.65) cm. The mean (SD) waist circumference was 81.16 (9.14) cm in men and 85.62 (11.74) cm in women. The overall mean waist circumference among men increased by age. Like in the case of body mass index, women 45–60 years had the highest mean waist circumference.

Based on the International Diabetes Federation (IDF) cut-off points for waist circumference (i.e., waist circumference >90 cm for men and >80 cm for women) [28], the overall prevalence of increased waist circumference was about 38.8 %. Women had an increased waist circumference prevalence of 66 %. While men had a 16 % prevalence of increased waist circumference. Using the American Heart association’s (AHA) definition of abdominal obesity (i.e., waist circumference > 102 cm for men and >88 cm for women), the prevalence of abdominal obesity was 18.8 %, about 38.2 % in women and 2.4 % in Men. By both standards, those above 60 years of age had highest prevalence of abdominal obesity (49.6 % using IDF and 24.9 % using AHA standards).

### Waist to hip ratio (WHR)

The mean (SD) hip circumference was 90.4 (8.7) cm in men and 98.5 (11.6) cm in women. Waist to Hip Ratio, the ratio of the circumference of the waist to that of the hip, was also used as a measure of obesity. In this study, the mean (SD) WHR of the study population was 0.89 (0.67) in men and 0.87 (0.76) in women. WHR had an increasing pattern by age of the respondents.

The World Health Organization’s (WHO), cut off point of WHR is >0.90 for men and >0.85 for women [29]. Based on this standard, the overall prevalence of increased WHR in the study population was 54.4 %, nearly 60 % in Women and 50 % in Men. The prevalence high WHR increased by age.

### Waist to height ratio (WHtR)

Waist to Height Ratio, the person’s waist circumference divided by the person’s height, is a measure of the distribution of body fat. The overall mean (SD) WHtR in this study was 0.51 (0.07), that is 0.49 (0.05) in men and 0.54 (0.07) in women. The mean WHtR among those less than 45 years, 45–60 years and above 60 years of age were 0.49, 0.52, and 0.53, respectively. Using 0.5 as a cut-off point for WHtR, the overall prevalence of increased WHtR was 49.73 %. This was 67.9 % in women and 34.3 % in men. The higher the age the higher the WHtR. Only 559 (11.21 %) had waist to height ratio of greater than 0.60 (Table 1).

### Relationship among the anthropometric indices

There exists a positive correlation among the four anthropometric markers of cardio-metabolic conditions. The overall inter-index correlation was +0.44. The average inter-index correlation for the indices of centeral obesity (WC, WHR, and WHtR) was +0.58. As shown in Table 3, the strongest correlation was between WC and WHtR (>0.93). A remarkable level of correlation was also observed between BMI and WHtR. The correlation between BMI and WC was also strong, especially among women. The correlations of WHR with waist circumference and WHtR ratio were moderate. WHR generally had lower correlation with the other anthropometric indices Table 2.

**Table 2:** Correlations among anthropometric markers of cardio-metabolic diseases

	BMI-WHR	BMI-WC	BMI-WHtR	WHR-WC	WHR-WHtR	WC-WHtR
Overall	0.12	0.69	0.75	0.44	0.38	0.93
Men (all)	0.27	0.58	0.66	0.51	0.52	0.92
>60	0.26	0.57	0.61	0.48	0.49	0.93
45–60	0.28	0.61	0.67	0.48	0.49	0.93
<45	0.23	0.55	0.65	0.48	0.49	0.91
Women (all)	0.15	0.74	0.75	0.49	0.49	0.95
>60	0.09	0.71	0.73	0.32	0.34	0.94
45–60	0.12	0.75	0.77	0.46	0.45	0.95
<45	0.15	0.74	0.77	0.50	0.49	0.95

For further understanding of the correlation matrix and better explanation of the existing correlations, we did a correlational analysis among height, weight, waist circumference and hip circumference measurements. Three relationships were highly correlated:. waist and hip circumference as well as body weight with both waist and hip circumference. The correlation between waist circumference and hip circumference was high at 0.78 indicating the drawbacks of WHR in assessing obesity when both waist circumference and hip circumference co-vary.

### Concordance among anthropometric indices

As indicated in the above sections, the prevalence of above normal body composition was 28.3 % by BMI, 38.9 % by hWC, 49.7 % by WHtR and 54.4 % by WHR. As presented in Table 3 analysis of the agreement between these main measures in classifying above normal body composition showed that there exists a substantial agreement between WC and WHtR. It was also observed that only a slight agreement exists between BMI and WHR Table 3.

**Table 3:** Agreement (Kappa) among anthropometric markers for classifying of obesity

	Male	Female	Both sexes	Agreement
BMI-WC	85.66 %(0.47)	70.66 %(0.44)	78.80 %(*k* = 0.53)	Moderate agreement
BMI-WHR	52.27 %(0.14)	52.94 %(0.09)	55.30 % (*k* = 0.14)	Poor agreement
BMI-WHtR	76.09 %(0.39)	69.95 %(0.43)	73.29 % (*k* = 0.46)	Moderate agreement
WC-WHR	60.40 %(0.21)	68.33 %(0.33)	64.03 %(*k* = 0.29)	Fair agreement
WC-WHtR	81.17 %(0.52)	90.37 %(0.78)	85.40 % (*k* = 0.71)	Substantial agreement
WHR-WHtR	67.81 %(0.35)	69.00 %(0.34)	68.36 % (*k* = 0.37)	Fair agreement

### Discordances among anthropometric markers

Of the total study population, 1739 (35.4 %) had normal BMI but increased WHR signaling possible presence of central/android obesity alone. This was high in men 1108 (38.1 %) than in women 721 (32.2 %). This may be a sign of ‘apple’ shaped body structure. In this analysis, 459 (9.3 %) of the study population had normal WHR but increased BMI indicating either the presence of peripheral obesity (i.e., higher fat accumulation in peripheral body parts) or a more muscular body architecture (i.e., higher weight due to higher muscle mass).

Moreover, 510 (10.2 %) of the respondents had normal WHR but increased WC indicating either a higher hip circumference (“pear’ shaped body – gynacoid obesity) or a proportional increase of fat accumulation both in the abdomen and lower half of the body. Of the study population, 1296 (25.8 %) had normal WC but high WHR indicating the presence of low muscle mass at the hip.

It was also found that 633 (12.7 %) study participants had high WHtR but normal WC. This may be due to short low stature that increased the WHtR. Comparison between WHR and WHtR showed that 903 (18.2 %) study participants had high WHR but normal WHtR while 670 (13.5 %) individuals had high WHtR but normal WHR. These discordances may be due to the relative size of hip circumference as compared to height.

Furthermore, analysis of the discordance between WC and BMI showed that 790 (16 %) individuals had high WC but normal BMI again signaling presence of central obesity. More than one-third, 1739 (35.4 %), of the study population had high WHR but normal BMI due to high WC relative to body weight.

### Combination of anthropometric indices

Combination of the four anthropometric indices showed that 829 (16.9 %) of the respondents had increased levels in all four indices. More than two third, 3448 (70 %) had above normal body composition in at least one of the four indices. About one-fifth of the respondents, 1055 (21.5 %), had increased levels in only one of the four indices: 73 (6.9 %) on BMI only, 35 (3.3 %) on WC only, 797 (75.5 %) on WHR only, and 150 (14.2 %) WHtR only. One-fifth of the respondents (20.1 %) had increased levels on any three of the four indices. Among these triads, the most common were WC-WHR-WHtR (57.4 %) and BMI-WC-WHtR (32.8 %).

### Association with cardio-metabolic risk

In this study, the weighted prevalence of hypertension (with hypertensive patients under treatment included regardless of their current blood pressure), hyperglycemia (with known diabetes patients included regardless of their blood sugar), and hypercholesterolemia were 7.0, 2.5 and 10.3 %, respectively. The analysis of the relationships between each of the four anthropometric indices with hypertension, hyperglycemia and hypercholesterolemia (after adjustment for age and sex), showed that WC was a stronger predictor of hypertension and hyperglycemia, with odds ratio of 2.27 (95 % CI: 1.80, 2.86) and 4.07 (95 % CI: 2.70, 6.15) as compared to other anthropometric indices. As indicated in Table 4, WHR is the strongest predictor of hypercholesterolemia, with odds ratio of 1.83 (95 % CI:1.52, 2.22), as compared to the other three indices. All anthropometric indices, except WHR, which better predicted hypercholesterolemia, were better predictors hyperglycemia as compared to their relationship with other cardio-metabolic markers Table 4.

**Table 4:** Binary relationship between anthropometric indices and cardio-metabolic makers (odds ratio with 95 % confidence intervals; adjusted for age and sex)

	Hypertension	Hyperglycemia	Hypercholesterolemia
BMI	2.13 (1.77, 2.58)	2.48 (1.79, 3.43)	1.52 (1.26,1.84)
WC	2.27 (1.80, 2.86)	4.07 (2.70,6.15)	1.38 (1.09, 1.74)
WHR	1.55 (1.28, 1.87)	1.61 (1.15, 2.25)	1.83 (1.52, 2.22)
WHtR	1.73 (1.43, 2.11)	2.43 (1.70, 3.48)	1.56 (1.29, 1.89)

## Discussion

In this study, we have found that the prevalence of above normal body composition was 28.3 % by BMI, 38.9 % by WC, 49.7 % by WHtR and 54.4 % by WHR. About 17 % of the study population had increased levels in all the four anthropometric indices. The overall inter-index correlation was found to be moderate at +0.44. The findings had also demonstrated that WC was the strongest predictor of hypertension and hyperglycemia while WHtR was the strongest predictor of hypercholesterolemia.

Even though there exists persistently high prevalence of under-nutrition, the prevalence of overweight and obesity is consistently increasing in sub-Saharan Africa (SSA). More recently, significant increases in the rates of overweight and obesity are being reported in SSA, especially among women and people living in urban areas. The findings from our study are consistent with this fact. As prevalences of overweight and obesity rise in SSA, rates of cardio-metabolic diseases are expected to rise [30].

In Kenya, especially in slum population, there is paucity of evidence about the magnitude of overweight and obesity. This study provided good estimates of prevalence of overweight and obesity measured by each of the four anthropometric indices as well as a combination of them. The prevalence of over normal body composition varied between 28.3 and 54.4 % based on the index used. Even using all the four indices together the prevalence of obesity in the study population is remarkable as nearly one-fifth of adults had above normal body composition. This calls for public health interventions in these urban poor population which were traditionally been considered as victims of under-nutrition.

BMI has traditionally been considered as the best standard in anthropometric measurement of obesity in adults. Most BMI standards were created based on population samples comprised of Caucasians and calculated using height and weight measurements. Research has now shown that BMI alone may not be an appropriate tool in determining obesity, due to differences in ethnicity and individual genetic make-up [31]. In our analysis, the discordance between BMI and other anthropometric indices was high signaling BMI as a weaker measurement of obesity compared to other indices.

A study conducted in Ghanian population indicated that WHR is one of the obesity measures most strongly linked with diabetes in this SSA population. In the same study, BMI was neither associated with diabetes in women nor in men. Thus, it may be useful for preventive strategies against type 2 diabetes to take into account WHR in addition to the conventional measure of BMI. In this region, women and men may equally benefit from public health efforts on the prevention of and the reduction in central obesity [32]. Our analysis also confirmed WC, not BMI, as the strongest predictor of hyperglycemia in slum population.

A study conducted in urban Cameroonian populations found high prevalences of overweight and obesity particularly above 35 years of age, and among women. Prevalence varied according to the measure used. The findings of this study highlighted the need to carry out further studies in Cameroonian and other Sub-Saharan African populations to provide appropriate cut-off points for the identification of people at risk of obesity-related disorders [33, 34]. Similarly, our study identified high prevalence of obesity and overweight among women and older age groups. The variations among the indices was also similar to that of Cameroon study.

Among Caucasians, WHtR and WC were found to be similar predictors of diabetes and CVD, both being stronger than, and independent of BMI. WHtR may be a more useful global clinical screening tool than WC, with a weighted mean boundary value of 0 · 5, supporting the simple public health message ‘keep your waist circumference to less than half your height’ [35]. In our study, the proportion of people with increased WHtR was high as compared to that of BMI. As under-nutrition during childhood could affect height, WHtR in our study population may overestimate the prevalence of obesity.

Robust statistical evidence from studies involving more than 300,000 adults in several ethnic groups, shows the superiority of WHtR over WC and BMI for detecting cardio-metabolic risk factors in both sexes. That study recommended WHtR to be considered as a screening tool [11]. Strengths of associations and discrimination statistics suggested that WHR was the best predictor of cardiometabolic events and mortality in patients with type-2 diabetes and BMI the worst [36]. As the cut-off points for WC and WHtR may vary across ethnic groups, this is something to be tested in Sub-Saharan African populations.

Evidence from an individual-participant meta-analysis of 82,864 participants from nine cohort studies showed that, in age- and sex-adjusted models only, BMI was related to CVD mortality but not in any other analyses. No major differences were revealed in the discrimination capabilities of models with BMI, WC or WHR for cardiovascular or total mortality outcomes. In the same study, measures of abdominal adiposity, but not BMI, were related to an increased risk of CVD mortality. No difference was observed in discrimination capacities between adiposity markers [37]. In our study we reported WC and WHR as the strongest predictors of Cardiometabolic markers.

The average inter-index correlation among the anthropometric indices in this study was moderate at +0.44 though some binary correlations were very high. Analysis of correlations between the indices was essential in this study for two reasons. The first one was related to ‘co-occurrence’ of different forms of overweight/obesity in an individual. For instance, the strong correlation between BMI (which measures generalized obesity) and WC (which measures central/abdominal obesity) indicated that these two forms of obesity tends to co-occur. The second reason was related to the ‘measurement’ of obesity in an individual. For instance, the strong correlation between WC (an absolute measure of waist) and WHtR (a relative measure), indicated that the two measurements are strongly related to each other. We know that WC is the numerator in WHtR. The strong correlation suggested that, in the study population, correcting WC for height had a minimal correction effect in qualifying the indicator.

However, there are some limitations associated with this study. First, there was no gold standard measure to compare all indices. As it is, it could be that BMI is different because it used quite different parameters that measure general obesity as compared to the other indices which all share one parameter (waist circumference) and measures central/abdominal obesity. So it is difficult to know which one of these measures is the best predictor of above normal body composition on the study setting. Second, some of the cut-off points were set for a different population groups and may not necessarily fit well with our study populations. Finally, this study is a cross-sectional study that lacks short of establishing the relationship between the different measures of body composition in an individual.

## Conclusions

The weighted prevalence of above normal body composition was 21.6 % by body mass index, 28.9 % by waist circumference, 45.5 % by waist to hip ratio, and 38.9 % by waist to height ratio. Combination of the four indices shows that 829 (16.9 %) of the respondents had increased levels in all four indices. More than two third of the study population, 3448 (70 %) had increased levels in at least one of the four indices. There was a modest level of correlation among the four adiposity markers. On the other hand, there exists a remarkable level of discordance among the four anthropometric indices. Moreover, this study had found WC as the strongest predictor of hypertension and hyperglycemia; and WHR as the strongest predictor of hypercholesterolemia. It is evident from the findings that while SSA has traditionally faced with issues of under-nutrition, this paper illustrates that it is also faced with the increasing prevalence of overweight and obesity. As it has been shown that some overweight and obese people eat a lot of nutrient poor foods and are in fact suffer from under-nutrition, in reality, SSA is facing with both under-nutrition and obesity.

Despite a consistently higher prevalence of above normal body composition in all anthropometric indices using the commonly used cut-off points, there was high level of variation between these anthropometric markers in estimating prevalence of above normal body composition. This may be partly due to the differences in what the indices actually measure. This could also be due to the limitations associated with the applicability of cut-off points in our study populations. We recommend further study to re-examine the applicability of these cut-off points in Africa populations. The use of adiposity index and other measures of body composition such as visceral and subcutaneous fat mass in addition to BMI in order to detect subjects with cardio-metabolic risk are also recommended.

## Abbreviations

AHA: American heart association
BMI: Body mass index
CVD: Cardiovascular diseases
GBD: Global burden of disease
HC: Hip circumference
IDF: International diabetes federation
NCDs: Non-communicable diseases
NHUDSS: Nairobi urban health and demographic surveillance system
PBF: Percentage of body far
SSA: Sub-Saharan Africa
WC: Waist circumference
WHO: World health organization
WHR: Waist to hip circumference ratio
WHtR: Waist circumference to height ratio
